# LncRNA growth arrest‐specific transcript 5 targets miR‐21 gene and regulates bladder cancer cell proliferation and apoptosis through PTEN

**DOI:** 10.1002/cam4.2664

**Published:** 2020-02-18

**Authors:** Dong Chen, Yihong Guo, Yaqiu Chen, Qiaonan Guo, Junyi Chen, Yining Li, Qiuping Zheng, Minyao Jiang, Ming Xi, Lu Cheng

**Affiliations:** ^1^ Department of Urology The Second Affiliated Hospital of Fujian Medical University Quanzhou China; ^2^ Department of Oncological Surgery The Second Affiliated Hospital of Fujian Medical University Quanzhou China; ^3^ Department of Urology Huadu District People's Hospital Southern Medical University Guangzhou China; ^4^ Department of Clinical Laboratory Huadu District People's Hospital Southern Medical University Guangzhou China

**Keywords:** bladder cancer, growth arrest‐specific transcript 5, long noncoding RNAs, miR‐21

## Abstract

The aim of this study was to investigate the mechanism by which growth arrest‐specific transcript 5 (GAS5) regulates bladder cancer cells. Bladder cancer samples were collected and tested for experiment. Dual‐luciferase reporter assay was used to verify the downstream target genes for GAS5 and miR‐21. The expression level of GAS5 was decreased and that of miR‐21 was increased, indicating a negative correlation between the two. Patients with high GAS5 level and low miR‐21 level had relatively longer survival rates. GAS5 inhibited bladder cancer cells proliferation and promoted apoptosis, and miR‐21 had the opposite effects. MiR‐21 was a direct target for GAS5, whereas phosphatase and tensin homolog (PTEN) was a direct target gene of miR‐21. Low expression of miR‐21 could reverse the proliferative and antiapoptotic effects caused by GAS5 silencing. High levels of GAS5 and low levels of miR‐21 might be associated with a higher survival rate in bladder cancer patients. GAS5 could exert antiproliferative and proapoptotic effects on bladder cancer cells through miR‐21 and PTEN.

## Graphical abstract

In bladder cancer, the expression level of growth arrest‐specific transcript 5 (GAS5) was decreased and the expression level of miR‐21 was elevated, and high levels of GAS5 and low levels of miR‐21 might be associated with higher survival. MiR‐21 was a target of GAS5, and PTEN was a target gene of miR‐21. GAS5 could inhibit the proliferation of bladder cancer cells and promote apoptosis by regulating PTEN through miR‐21.

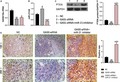

## 1 INTRODUCTION

Bladder cancer is a common malignant tumor, and it has the fourth highest incidence in men among malignant tumors.^1^ The main method of treating bladder cancer is endoscopic resection. However, surgical treatment has limited effect on advanced bladder cancer, and clinical study have found that bladder cancer recurrence and metastasis occur to about half of patients after radical surgery.^2^, ^3^ At present, the pathological mechanism of bladder cancer is still unclear, which brings up limitations to the treatment of bladder cancer.

Long noncoding RNAs (LncRNAs) play an important role in many life activities such as epigenetic, cell cycle, and cell differentiation.^4^, ^5^, ^6^ The research on the role of LncRNAs in tumors is a hotspot in genetic research. Studies have confirmed that LncRNAs played an impotent role in tumor cell cytolergy.^7^, ^8^, ^9^ Growth arrest‐specific transcript 5 (GAS5), which located at chromosome 1q25, has an inhibitory effect on tumors.^10^ In recent years, study showed that GAS5 was underexpressed in bladder cancer and promoted cell proliferation by regulating CDK6.^11^ However, the role of GAS5 in bladder cancer and related mechanisms is rarely studied.

Recent study has shown that LncRNAs interact with microRNAs (miRNAs), and some LncRNAs act by modulating miRNAs.^12^ miRNAscan regulate protein expression by inhibiting or inducing the degradation of messenger RNAs (mRNAs) by specifically identifying the 3′ untranslated region (UTR) of the mRNAs.^13^, ^14^

Studies have shown that miR‐21 is associated with prognosis of colorectal cancer, esophageal cancer, and hepatocellular carcinoma.^15^, ^16^, ^17^ Recent study has also shown that miR‐21 participates in the development and progression of bladder cancer through transforming growth factor‐beta variants or PPP2R2A/ERK.^18^ In recent years, study has found that GAS5 can inhibit metastasis of liver cancer cells by inhibiting miR21 in hepatocellular carcinoma.^19^

Therefore, this study mainly analyzed the relation between GAS5 and miR‐21 and prognosis in bladder cancer as well as their roles in cell cytolergy of bladder cancer cells and the related mechanisms. Our research provides new possibilities for clinical treatment of bladder cancer.

## 2 MATERIALS AND METHODS

### 2.1 Patients and samples

Thirty‐five bladder cancer tissues from 22 males and 13 females who aged 43‐80 years old (at an average age of [54.53 ± 4.23] years old) were collected from March 2015 to March 2016. All patients enrolled were diagnosed with bladder cancer by pathology and did not have other malignancies. The patients were firstly be diagnosed and did not receive radiotherapy, chemotherapy, or immunotherapy. The tissues were stored at −80°C for subsequent testing. The GAS5 and miR‐21 mRNA levels in each sample were detected, and U6 and 18s‐rRNA were used as internal controls, respectively. Correlation analysis was performed on the levels of GAS5 and miR‐21. Survival analysis was performed on patients with different GAS5 and miR‐21 expression levels. This study was approved by the Ethics Committee and an informed consent was signed by all patients.

### 2.2 Cells culture and transfection

SV‐HUC‐1, HTB‐9, J82, UM‐UC‐3, and T24 cells (ATCC) were cultured in RPMI 1640 medium containing 10% fetal bovine serum.

The GAS5 coding sequences were subcloned into pcDNA3.1 (Genechem) to construct pcDNA expression vectors. GAS5 and siGAS5 (GenePharma) transfections were performed using Lipofectamine 2000 (Invitrogen), and the cells were harvested after 24 hours.

The miR‐21 mimic (RiboBio) and locked nucleic acid (LNA)‐anti‐miR‐21 (Exiqon) were used to upregulating or downregulating miR‐21 levels.

### 2.3 Dual‐luciferase reporter assay

The targets gene of GAS5 and miR‐21 were predicted on the webset. The relation between phosphatase and tensin homolog (PTEN) downregulation and overall survival rate of bladder cancer patients was investigated by the cancer genome atlas (TCGA). Bladder cancer cells were transfected with the luciferase reporters together with GAS5 and miR‐21, or GASS‐3′‐UTR‐mutant (mut) and miR‐21 by Lipofectamine 2000. Forty‐eight hours after transfection, luciferase experiment was performed using a dual‐luciferase reporter assay kit (Promega).

### 2.4 Cell counting kit‐8 ASSAY

The Cell counting kit‐8 (CCK‐8) assay purchased from Tongren (Japan) was applied to test the cell viability. The optical density values at 450 nm were measured (ELX 800; Bio‐Teck).

### 2.5 Flow cytometry

Flow cytometry was used to detect cell apoptosis and the kits were purchased from BD Pharmingen. 1 × 10^6^ cells were washed with PBS at 4°C and resuspend to a concentration of 4 × 10^5^ cells/mL. Flow cytometer and supporting kits (Becton Dickerson) were applied to analysis the cell apoptosis. Flow cytometry was also applied to detect cell cycle.

### 2.6 Mice modeling and sample collection

BALB/c mice (Laboratory Animal Cecter) were randomly divided into three groups (n = 3) as follows: negative control (NC) group, GAS5 siRNA group and GAS5 siRNA + miR‐21 inhibitor group (n = 3). The cells (×10^6^) were injected subcutaneously and weighed the tumors when the average tumor diameter reached about 1.0 cm in the NC group.

Streptavidin‐perosidase staining tumor samples specimens were cut in 4‐μm‐thick sections and deparaffinezed. Repair and containment were performed using 0.01 mol/L citrate buffer and peroxidase, respectively. Primary and secondary antibodies were added according to the kit. Finally, DAB staining was added for 5 minutes and observed under a microscope.

### 2.7 Quantitative real‐time polymerase chain reaction

The levels of mRNA were detected by quantitative real‐time polymerase chain reaction (qRT‐PCR). Total RNA of cells were extracted by Trizol (Invitrogen) and transferred to cDNAs by iScriptTMcDNA Synthesis Kit (Bio‐Rad). Quantitative real‐time polymerase chain reaction was performed with the qRT‐PCR kit (TaKaRa). Procedures were set as the following: 2 minutes at 95°C for hot start, 40 cycles of 15 seconds at 95°C, 30 seconds at 60°C, and 60 seconds at 72°C. Primers (Table 1) were obtained from Genewiz (Suzhou). The formula $2^{-\Delta \Delta C_{T}}$ was used to analyze the mRNA expression levels.

**Table 1:** The sequences of primers

Primer name	Sequence (5′‐3′)
GAS5‐forward	CTTGCCTGGACCAGCTTAAT
GAS5‐reverse	CAAGCCGACTCTCCATACCT
18s‐forward	GTAACCCGTTGAACCCCATT
18s‐reverse	CCATCCAATCGGTAGTAGCG
miR‐21‐forward	ACACTCCAGCTGGGTAGCTTATCAGACTGA
miR‐21‐reverse	TGGTGTCGTGGAGTCG
U6‐forward	CGCTTCGGCAGCACATATACTAAAATTGGAAC
U6‐reverse	GCTTCACGAATTTGCGTGTCATCCTTGC
PTEN‐forward	GGGGTGGAACTGTGCACTAA
PTEN‐reverse	AGGCTTTGAAGGACAGCAGG
GAPDH‐forward	ATGGGGAAGGTGAAGGTCG
GAPDH‐reverse	GGGGTCATTGATGGCAACAATA

### 2.8 Western blot

Proteins were tested using Western blot. The cells were lysed and the total protein was collected. Total Proteins in samples were separated in a 10% sodium dodecyl sulfate‐polyacrylamide gel electrophoresis for 90 minutes at 100 V and further transferred to PVDF membrane by wet transfer method for 80 minutes at 90 V. Membrane was washed with PBST and blocked with 5% fat‐free milk for 2 hours. The primary antibodies (anti‐PTEN; Abcam, ab32199; anticleaved caspase‐3, ab13847; anti‐Bax, ab32503; anti‐Bcl‐2, ab692; anti‐Cyclin A, ab38; anti‐Cyclin D1, ab134175; anti‐cMyc, ab39688) were added at room temperature for 2 hours and incubated at 4°C for 12 hours. The corresponding secondary antibodies were added at room temperature for 1.5 hours. The blots were then scanned and filmed in a detector (ChemiDoc MP; Bio‐Rad). Image J was used to read the gray level.

### 2.9 Statistical analysis

One‐way ANOVA test was used for the statistical analysis in Graphpad 7.0 version. Each sample of cells was tested three times repetitively. *P* < .05 was considered significant.

## 3 RESULTS

### 3.1 In bladder cancer, the level of GAS5 was low, and miR‐21 level was high

**Figure 1:**
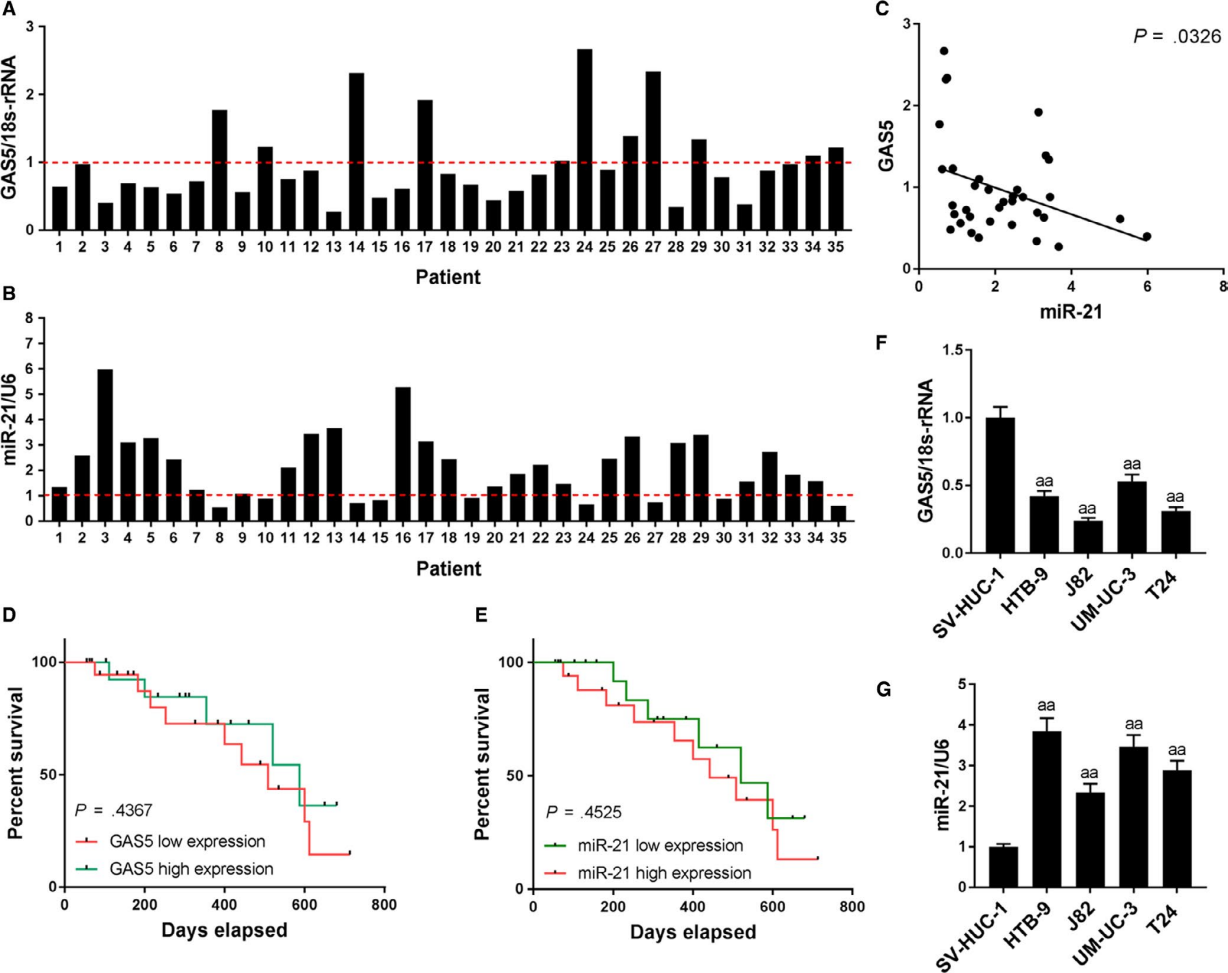
Expression characteristics of growth arrest‐specific transcript 5 (GAS5) and miR‐21 in bladder cancer tissues and cell lines. A‐C, The expression levels and correlations of GAS5 and miR‐21 in bladder cancer tissues from 35 patients. D and E, Comparison of survival rates of patients with different GAS5 and miR‐21 expression levels. F and G, Expression levels of GAS5 and miR‐21 in different bladder cancer cell lines. ^aa^ *P* < .01, versus SV‐HUC‐1

In 35 bladder cancer patients, 18 cases had high expression of GAS5, whereas 17 had low expression of miR‐21 (Figure 1A,B). Clinically relevant analysis showed that patients aged young, with small tumor size and adenocarcinoma, in low tumor stage, as well as without lymph node metastasis had higher GAS5 levels and lower miR‐21 levels (Table 2). Correlation analysis showed that the levels of GAS5 and miR‐21 in bladder cancer tissues were inversely related (*P* = .0326) (Figure 1C). Survival analysis showed a higher survival rate in bladder cancer patients with high GAS5 expression and low miR‐21 expression (Figure 1D,E). Cellular assay results also showed that in bladder cancer cells, GAS5 levels were low and miR‐21 levels were high (Figure 1F,G). This suggested that low GAS5 and high miR‐21 could be used to predict more severe bladder cancer and worse prognosis, and that high GAS5 level might be associated with low expression of miR‐21.

**Table 2:** Correlation between the levels of GAS5 and miR‐21 and the clinicopathological characteristics of bladder cancer

Characteristics	Number of patients	GAS5 low expression (<median)	GAS5 high expression (≥median)	*P* value	miR‐21 low expression (<median)	miR‐21 high expression (≥median)	*P* value
Number	35	17	18	<.05	17	18	<.05
Age (y)				<.05			<.05
≤60	19	6	13		12	7	
>60	16	8	8		5	11	
Tumor size (cm)				<.05			<.05
≤3.0	20	8	12		11	9	
>3.0	15	10	5		6	9	
Histology				<.05			<.05
Adenocarcinoma	21	9	12		11	10	
Squamous carcinoma	14	9	5		6	8	
Tumor stage				<.05			<.05
I	8	3	5		7	1	
II	14	7	7		6	8	
III	13	10	3		4	9	
Lymph node metastasis				<.05			<.05
No	20	7	13		14	6	
Yes	15	11	4		5	10	

### 3.2 High expression of GAS5 inhibited HTB‐9 cells proliferation and promoted apoptosis

**Figure 2:**
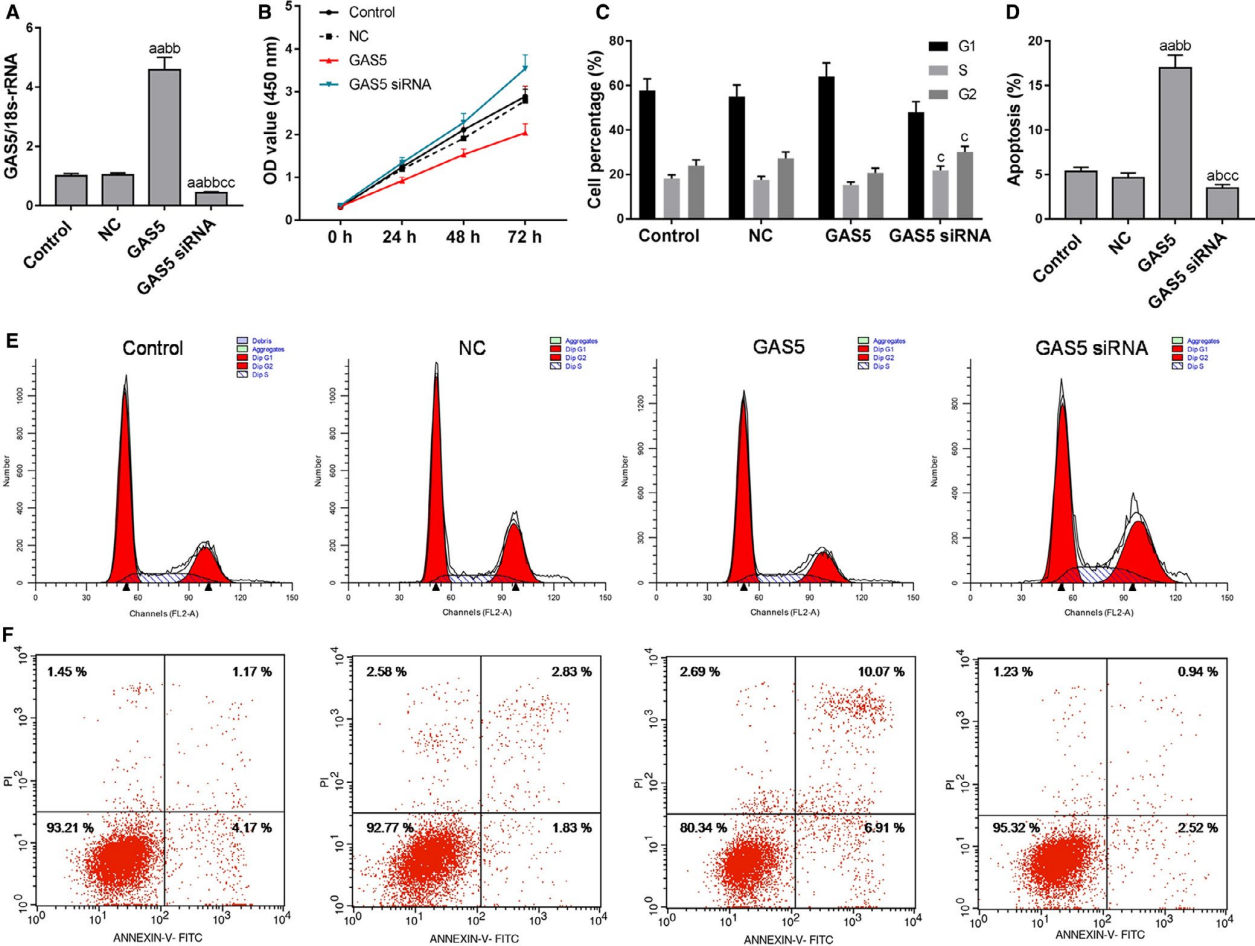
Effects of growth arrest‐specific transcript 5 (GAS5) on proliferation and apoptosis of bladder cancer cells. A, GAS5 mRNA was tested by quantitative real‐time polymerase chain reaction. B, Cell counting kit‐8 assay was applied to detected cell viability. C‐F, Flow cytometry was used to detect cell cycle and apoptosis at different GAS5 expression levels. ^a^ *P* < .05, ^aa^ *P* < .01, vs control group; ^b^ *P* < .05, ^bb^ *P* < .01, vs NC group; ^c^ *P* < .05, ^cc^ *P* < 0.01, vs GAS5 group

In order to study the effect of GAS5 on bladder cancer cells, GAS5 overexpression (GAS5 group) and low expression (GAS5 siRNA group) of HTB‐9 cells were constructed, and transfection efficiency was detected by qRT‐PCR. The results showed that GAS5 expression level in the GAS5 group was significantly higher than that in the control and NC groups (Figure 2A). This suggested the transfection experiment was successfully conducted. Next, proliferation and apoptosis of HTB‐9 cells with different GAS5 expression levels were detected, and the results showed that after overexpression of GAS5, the cell viability of HTB‐9 cells decreased and the apoptotic rate increased significantly (Figure 2B‐F). Downregulation of GAS5 expression levels promoted cell proliferation, increased the proportion of cells in the S and G2 phases and inhibited apoptosis (Figure 2B‐F). This suggested that GAS5 overexpression inhibited cell proliferation and promoted cell apoptosis.

### 3.3 miR‐21 was the target of GAS5

**Figure 3:**
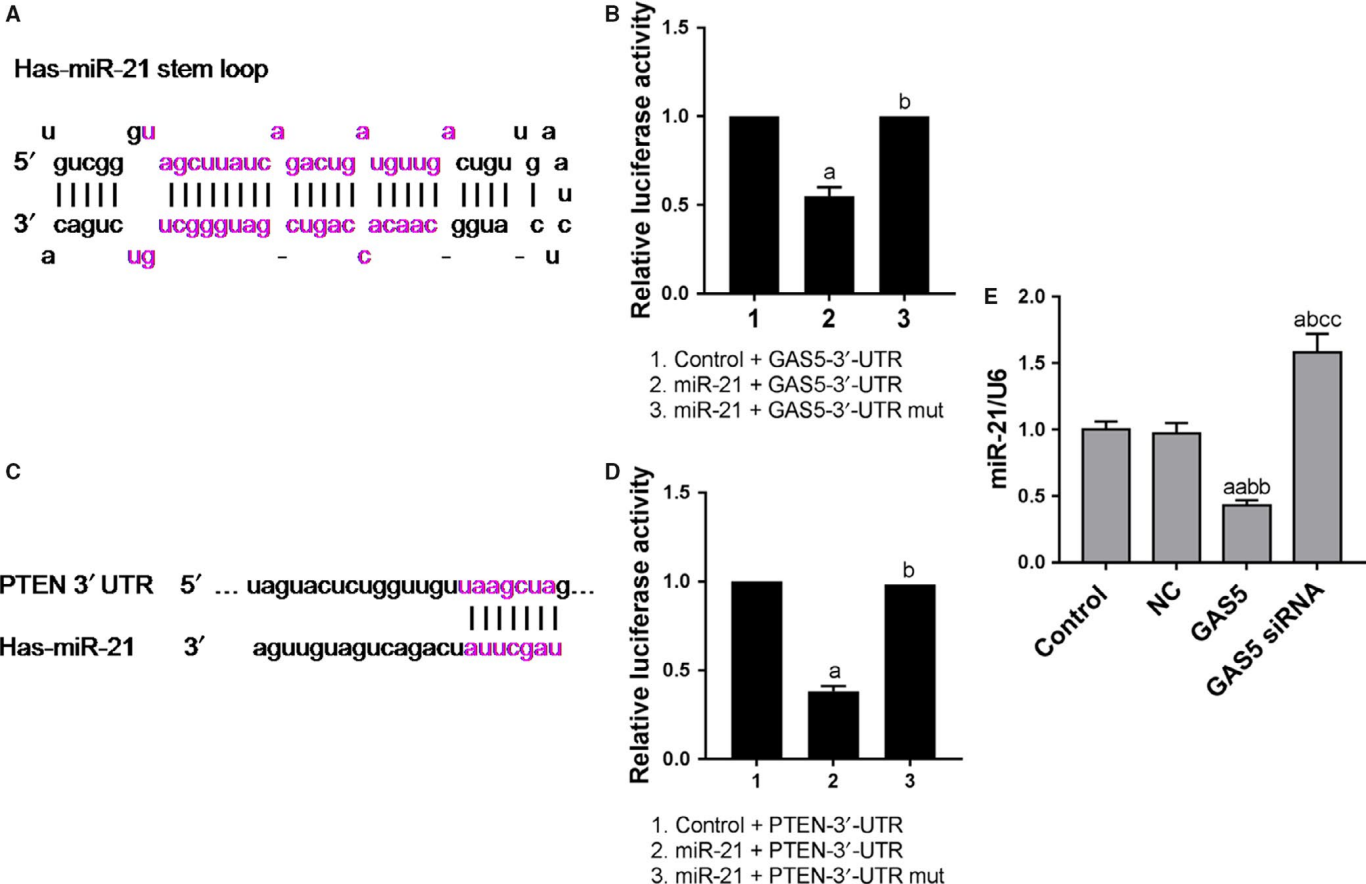
Prediction and validation of growth arrest‐specific transcript 5 (GAS5) potential target genes. A, The miR‐21 was predicted to be the potential target of GAS5. B, Dual‐luciferase reporter assay was used to verify that GAS5 directly regulated miR‐21 expression by 3′UTR. C, PTEN was a potential target for hsa‐miR‐21. D, Dual‐luciferase reporter assay was used to verify that miR‐21 directly regulated PTEN expression by 3′UTR. E, Quantitative real‐time polymerase chain reaction was applied to test the miR‐21 expression levels at different GAS5 expression levels. ^a^ *P* < .05, ^aa^ *P* < .01, vs control group; ^b^ *P* < .05, ^bb^ *P* < .01, vs NC group; ^cc^ *P* < .01, vs GAS5 group

Predicted by miRanda, homo sapiens miR‐21 (hsa‐miR‐21) was a potential target for GAS5 (Figure 3A) and it was testified by luciferase analysis (Figure 3B). In addition, PTEN was a potential target for hsa‐miR‐21 (Figure 3C) and it was testified by luciferase analysis (Figure 3D). Further studies showed that GAS5 overexpression downregulated miR‐21 level and GAS5 low expression increased miR‐21 level (Figure 3E). This demonstrated that miR‐21 was a direct target of GAS5, PTEN was a direct target for hsa‐miR‐21, and that low expression of GAS5 directly upregulated miR‐21 expression.

### 3.4 Low miR‐21 level inhibited HTB‐9 cells proliferation and promoted apoptosis

**Figure 4:**
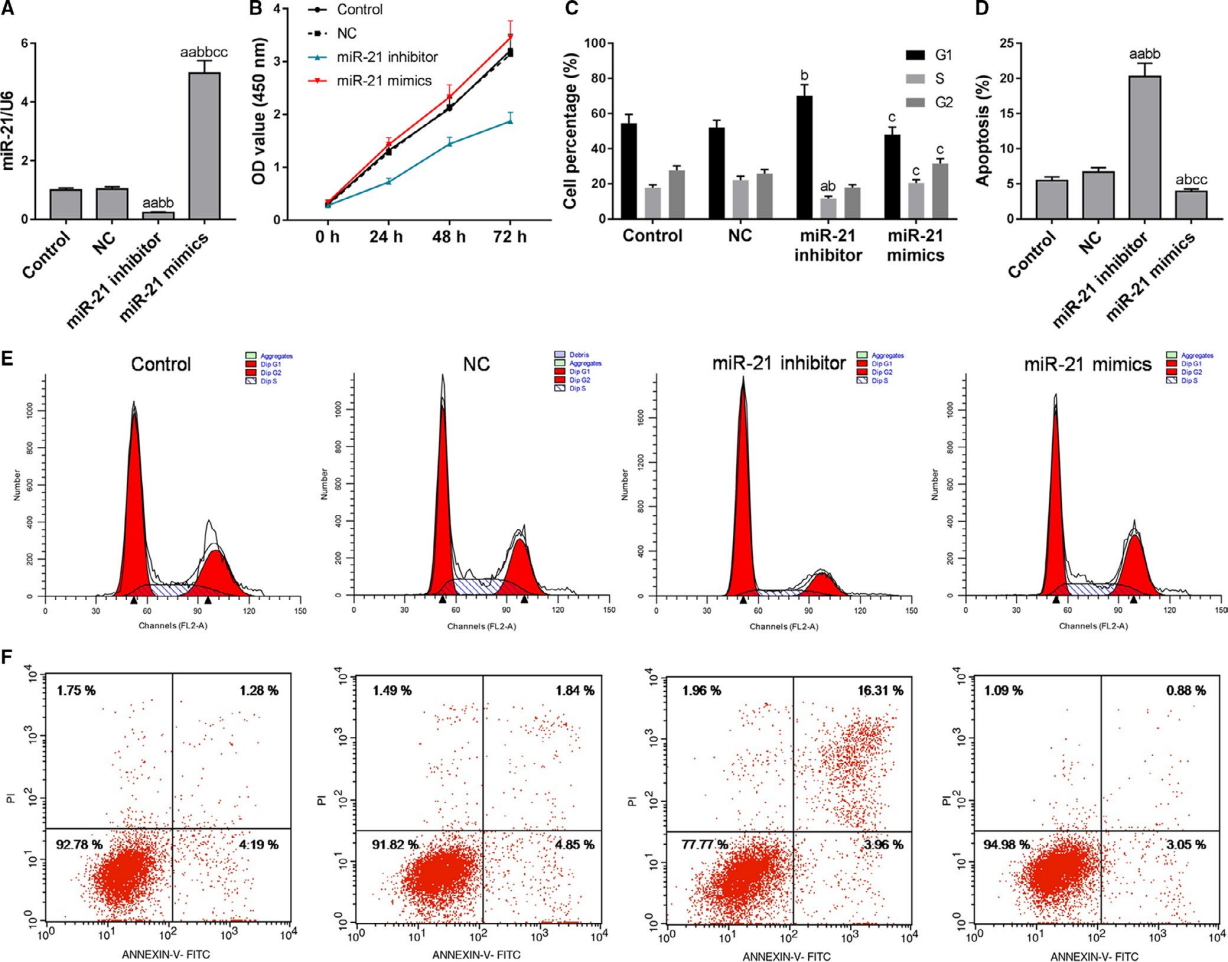
Effects of miR‐21 on proliferation and apoptosis of bladder cancer cells. A, miR‐21 was tested by quantitative real‐time polymerase chain reaction. B, Cell counting kit‐8 assay was applied to detected cell viability. C‐F, Flow cytometry was used to detect cell cycle and apoptosis at different miR‐21 expression levels. ^a^ *P* < .05, ^aa^ *P* < .01, vs control group; ^b^ *P* < .05, ^bb^ *P* < .01, vs NC group; ^c^ *P* < .05, ^cc^ *P* < .01, vs miR‐21 + inhibitor group

In order to study the effect of miR‐21 on bladder cancer cells, miR‐21 overexpression (miR‐21 mimics group) and low expression of HTB‐9 cells (miR‐21 inhibitor group) were constructed. miR‐21 mimics significantly increased miR‐21 level (Figure 4A). Proliferation and apoptosis of HTB‐9 cells with different miR‐21 levels were detected, and the data showed that the cell viability and cell percentage in S phase was decreased in the miR‐21 inhibitor group, whereas the apoptotic rate was increased (Figure 4B‐F). High level of GAS5 promoted cell proliferation, increased the cell percentage in the S and G2 phases and inhibited apoptosis (Figure 4B‐F). This suggested that low expression of miR‐21 had the effects of inhibiting cell proliferation and promoting cell apoptosis.

### 3.5 PTEN was the target of miR‐21

**Figure 5:**
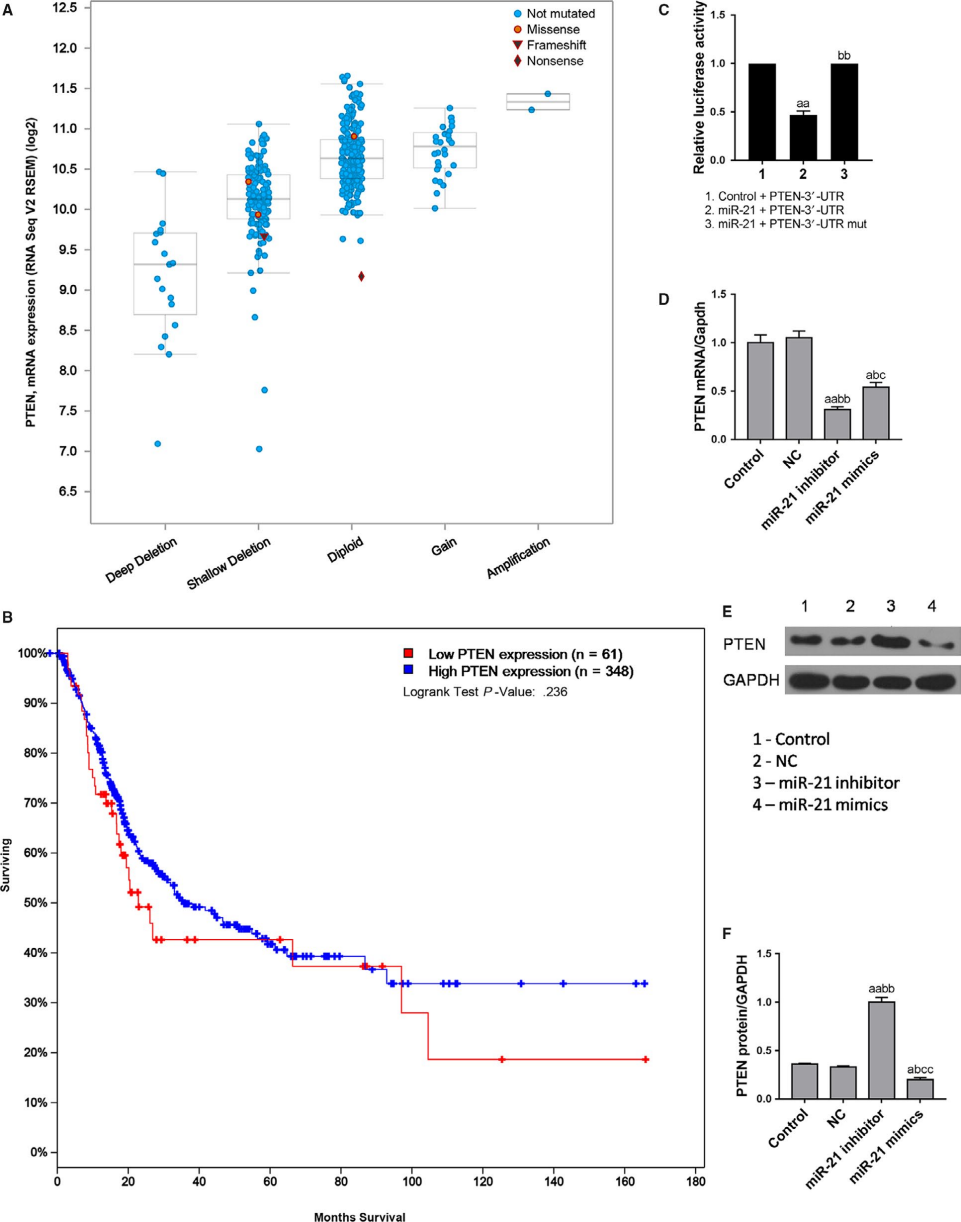
Prediction and validation of miR‐21 potential target genes. A and B, The relation between PTEN expression level and the survival of bladder cancer patients. C, Dual‐luciferase reporter assay was used to verify that miR‐21 directly regulated PTEN expression by 3′UTR. D‐F, Western blot and quantitative real‐time polymerase chain reaction were applied to test the miR‐21 expression levels at different miR‐21 expression levels. ^a^ *P* < .05, ^aa^ *P* < .01, vs control group; ^b^ *P* < .05, ^bb^ *P* < .01, vs NC group; ^c^ *P* < .05, ^cc^ *P* < .01, vs miR‐21 + inhibitor group

The miRanda, TargetScan, and Pictar sites predicted that the inhibitory gene PTEN was a target for miR‐21. According to TCGA data, low PTEN levels are associated with low survival rates (*P* = .236) (Figure 5A,B). Although not statistically significant, the difference between the two was obvious, and this might be due to our small number of cases of low PTEN expression in the database of the query. The dual‐luciferase reporter assay showed that PTEN was the target of miR‐21 (Figure 5C). Further studies showed that miR‐21 low expression upregulated PTEN level (Figure 5D,F). This demonstrated that PTEN was a direct target of miR‐21, and low expression of miR‐21 directly upregulated miR‐21 expression.

### 3.6 GAS5 regulated cell proliferation and apoptosis via miR‐21

**Figure 6:**
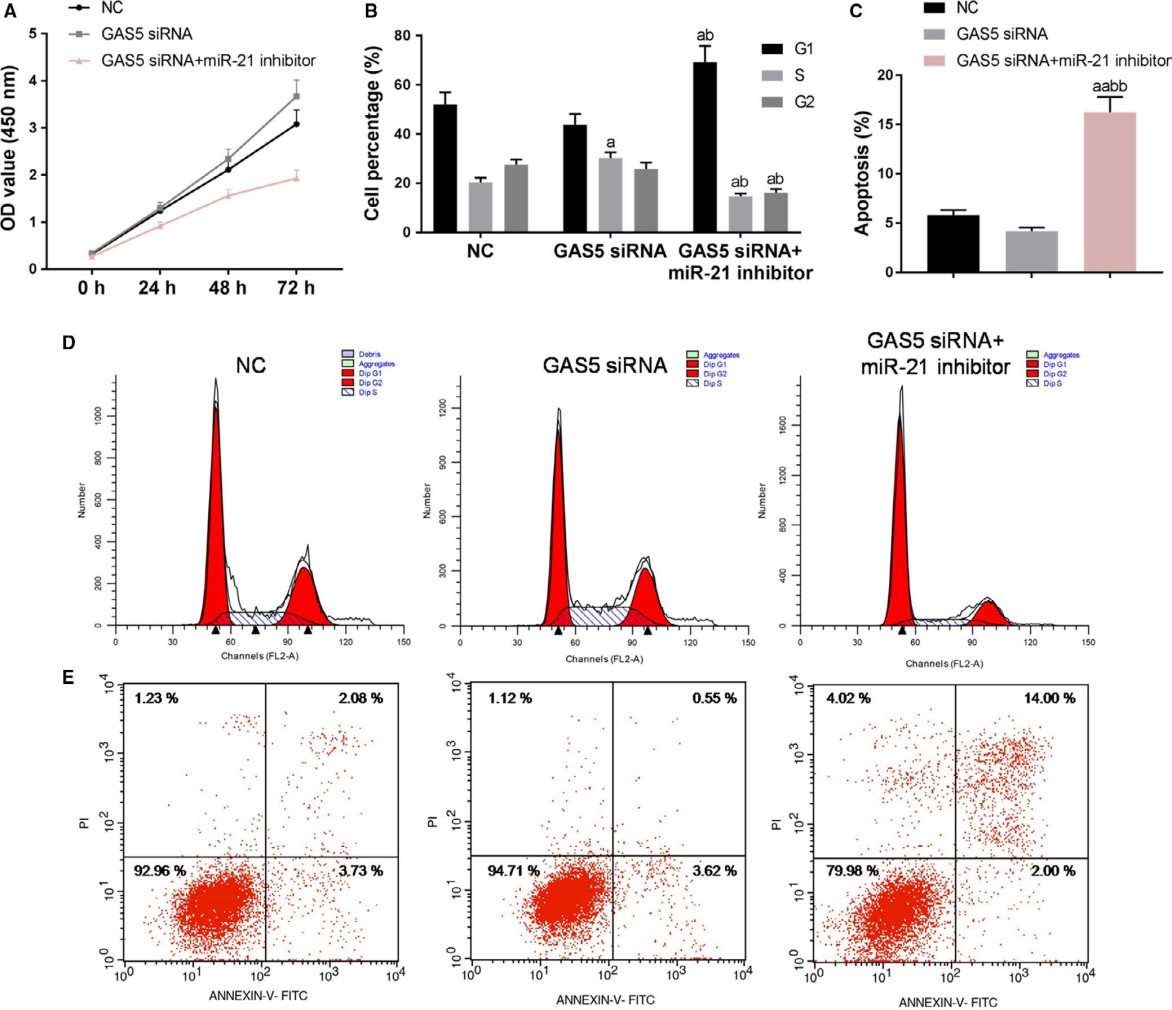
Effects of growth arrest‐specific transcript 5 (GAS5) and miR‐21 on proliferation and apoptosis of bladder cancer cells. A, Cell counting kit‐8 assay was applied to detected cell viability. B‐E, Flow cytometry was used to detect cell cycle and apoptosis at different miR‐21 expression levels. ^a^ *P* < .05, ^aa^ *P* < .01, vs NC group; ^b^ *P* < .05, ^bb^ *P* < .01, vs GAS5 siRNA group

To investigate the mechanism by which GAS5 and miR‐21 regulate proliferation and apoptosis of bladder cancer cells, NC group, GAS5 siRNA group and GAS5 siRNA + miR‐21 inhibitor group were constructed, respectively. We noted that the cell viability and the cell percentages in the S and G2 phases in the GAS5 siRNA + miR‐21 inhibitor group were significantly decreased and the apoptotic rate was increased (Figure 6A‐F), suggesting that GAS5 might regulate proliferation and apoptosis of HTB‐9 cells through miR‐21.

**Figure 7:**
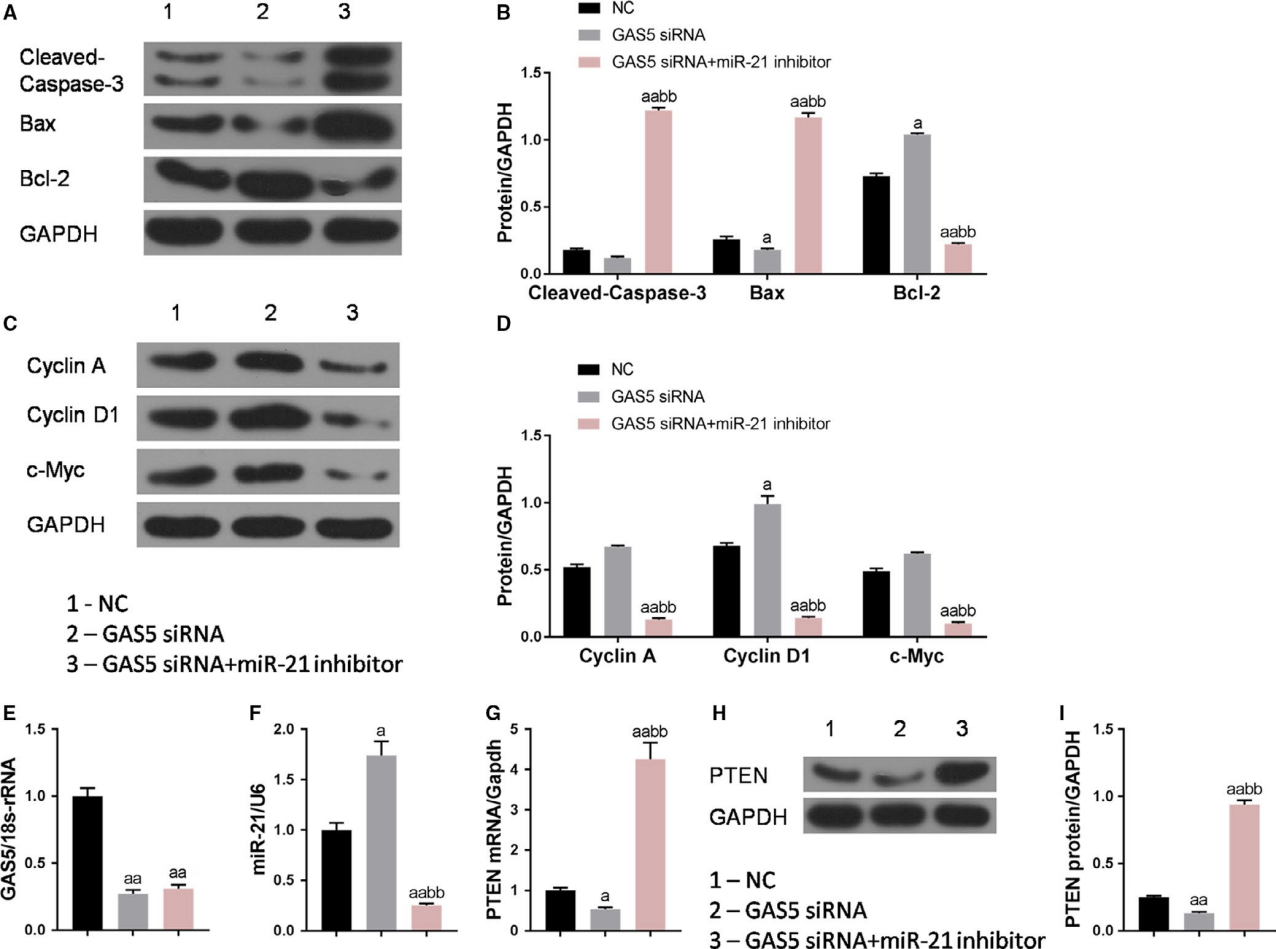
Expression characteristics of growth arrest‐specific transcript 5 (GAS5), miR‐21 and cell cycle‐related proteins and apoptosis‐related proteins. A‐D, Western blot was used to test the cell cycle‐related proteins and apoptosis‐related proteins expression levels. E and F, Quantitative real‐time polymerase chain reaction (qRT‐PCR) was applied to test the miR‐21 and GAS5 expression levels. G‐I, PTEN protein and mRNA levels were detected using Western blot and qRT‐PCR ^a^ *P* < .05, ^aa^ *P* < .01, vs NC group; ^bb^ *P* < .01, vs GAS5 siRNA group

To further investigate the mechanism of GAS5 regulating cell proliferation and apoptosis of HTB‐9 cells through miR‐21, Western blot was applied to detect the expression levels of apoptosis‐related proteins and cell cycle‐associated proteins in the three groups. The results showed that the Bax and Cyclin D in the GAS5 siRNA group were lower than those in the NC group, and that the Bcl‐2 was higher than that in the NC group. The cleaved caspase‐3 and Bax in the GAS5 siRNA + miR‐21 inhibitor group were significantly increased, whereas the Bcl‐2, Cyclin A, Cyclin D1, and cMyc were significantly decreased (Figure 7A‐D). This indicated that GAS5 might participate in the proliferation and apoptosis of bladder cancer cells by regulating miR‐21 and apoptosis‐related proteins and cell cycle‐associated proteins.

To further verify that GAS5 regulating cell proliferation and apoptosis through miR‐21 and PTEN, the expression levels of GAS5, miR‐21, and PTEN were detected in three groups. It showed that low level of miR‐21 had no significant effects on the expression level of GAS5, whereas low expression of GAS5 upregulated the expression level of miR‐21. Moreover, the levels of PTEN protein and mRNA in the GAS5 siRNA group were lower than those in the NC group, and that the levels of PTEN protein and mRNA in the GAS5 siRNA + miR‐21 inhibitor group were higher than those in the NC group and GAS5 siRNA group (Figure 7E‐I), indicating that low expression of miR‐21 upregulated the expression level of PTEN gene, whereas low expression of GAS5 could inhibit the expression of PTEN by upregulating miR‐21, thereby regulating the proliferation and apoptosis.

### 3.7 GAS5 could inhibit bladder tumor by miR‐21

**Figure 8:**
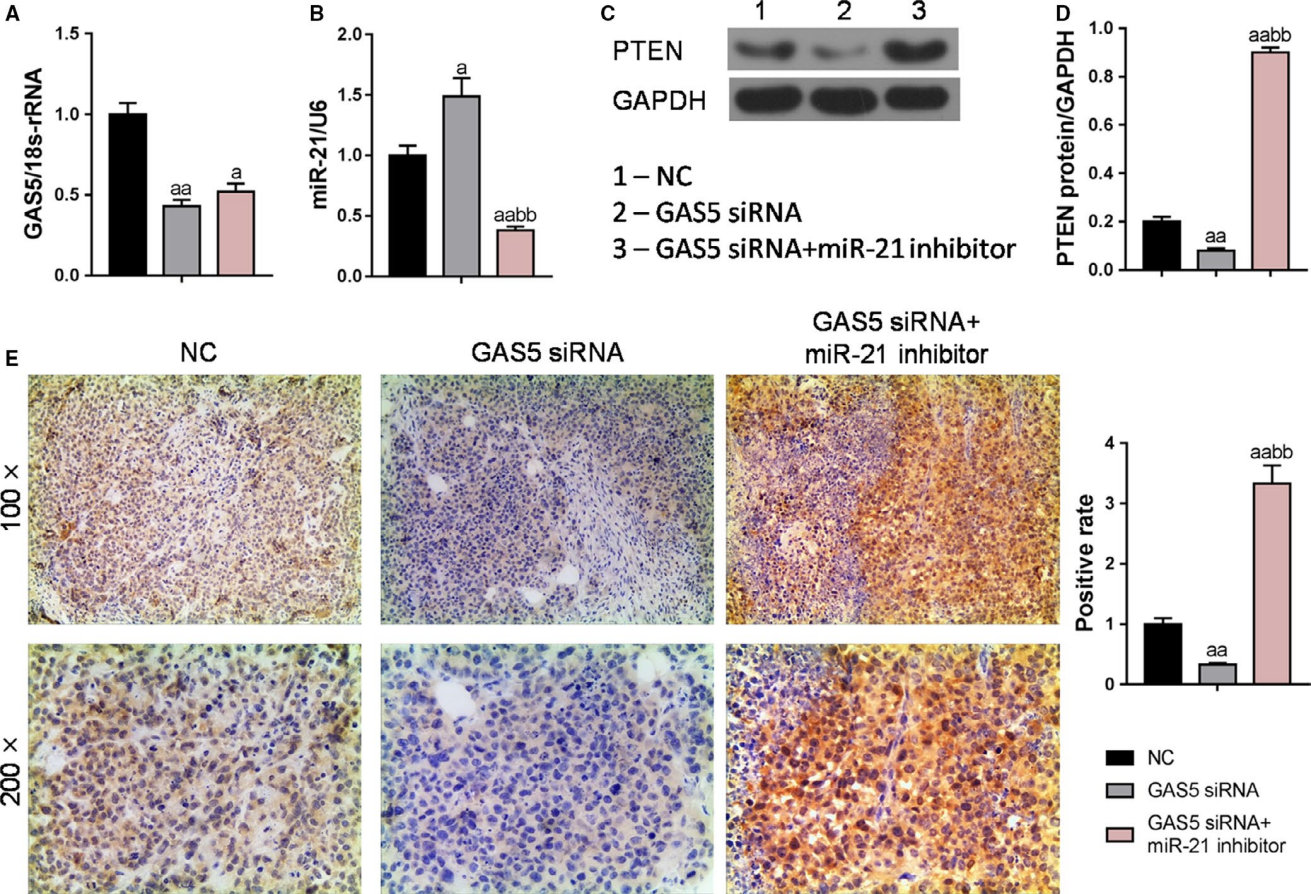
Expression characteristics of growth arrest‐specific transcript 5 (GAS5), miR‐21 and PTEN in bladder tumor tissue. A and B, Quantitative real‐time polymerase chain reaction was applied to test the miR‐21 and GAS5 expression levels. (C and D) PTEN protein levels were detected using Western blot. E, Streptavidin‐perosidase staining was used to detect the positive level of PTEN expression in mice subcutaneous bladder tumor. ^a^ *P* < .05, ^aa^ *P* < .01, vs NC group; ^bb^ *P* < .01, vs GAS5 siRNA group

**Figure 9:**
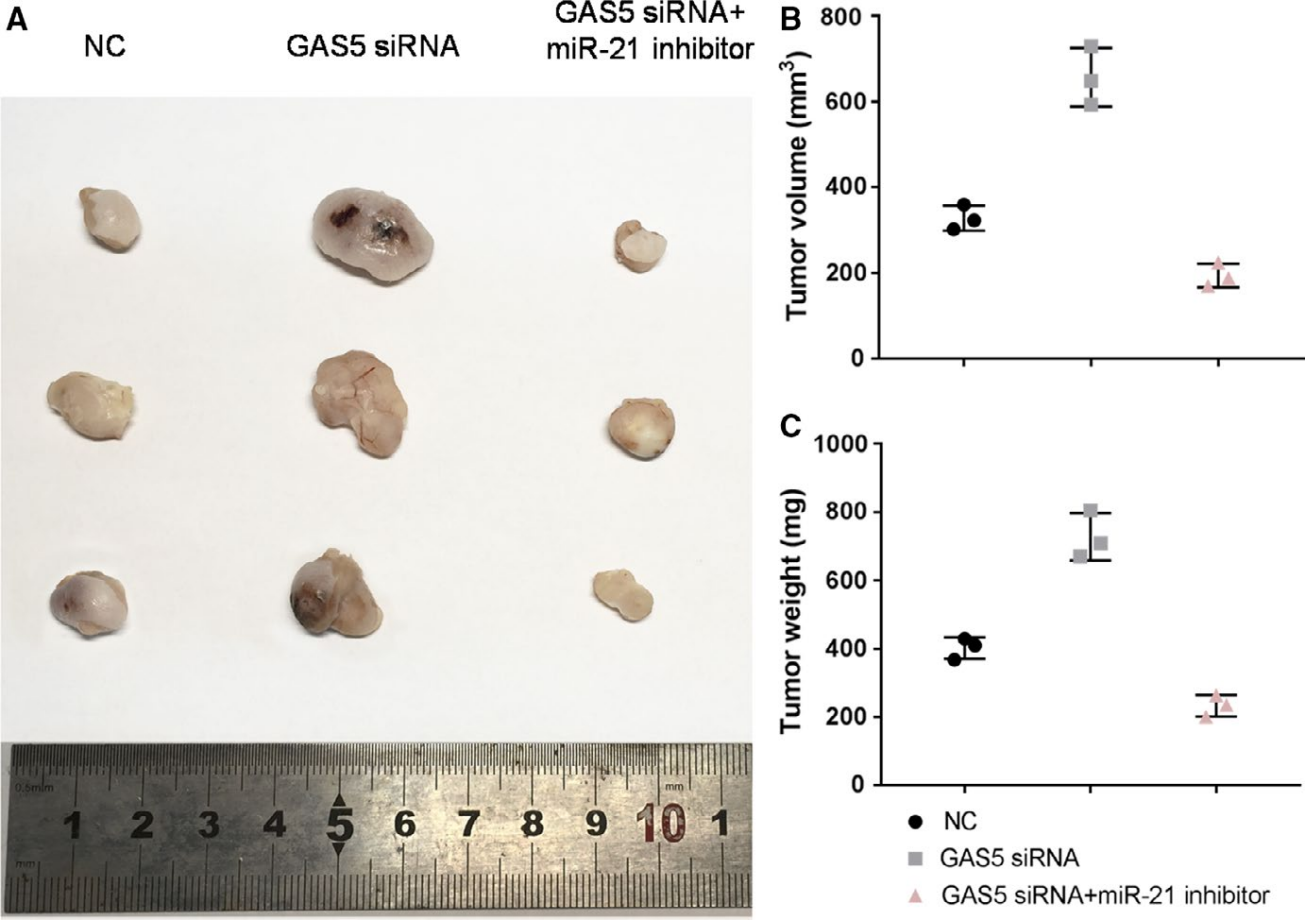
Inhibition of bladder tumor by growth arrest‐specific transcript 5 (GAS5) and miR‐21. A‐C, Comparison of size, volume, and weight of the bladder tumor in the three groups

The detection of tumor tissues revealed that the expression levels of GAS5, miR‐21 and PTEN were consistent with those in above cell experiments (Figure 8A‐D). Streptavidin‐perosidase staining results also showed that low expression of GAS5 inhibited the expression of PTEN protein, whereas silencing of miR‐21 upregulated the expression level of PTEN protein (Figure 8D‐E). Further studies showed that the tumor volume and weight in the GAS5 siRNA group were significantly higher than those in the NC group, whereas the tumor volume and weight in the GAS5 siRNA + miR‐21 group were significantly lower than those in the GAS5 siRNA group and NC group (Figure 9A‐C), meaning that low expression of miR‐21 could inhibit the growth of bladder cancer by upregulating the expression level of PTEN, and such a process was regulated by GAS5.

## 4 DISCUSSION

As a tumor marker, GAS5 is widely used in the prediction of a variety of tumor prognosis. It has been shown that the decrease in the expression level of GAS5 predicted a poor prognosis of gastric cancer, cervical cancer, and thyroid cancer.^20^, ^21^, ^22^, ^23^ Research on GAS5 in bladder cancer has gradually increased too. Study found that miR‐21 was one of the targets of GAS5, and the two had a negative correlation and contributed to the prognosis of cancers.^19^ To explore the relation between GAS5 and miR‐21 in bladder cancer, we first analyzed the relation between GAS5 or miR‐21 and bladder cancer patients' clinical data.

The results showed that in tumor tissues of patients with bladder cancer, GAS5 was generally lowly expressed but miR‐21 was highly expressed, and the two were negatively correlated to each other. After 2 years of follow‐up and survival analysis, it was found that patients with high levels of GAS5 and lower levels of miR‐21 had a higher survival rate. There was no uniform meaning because the number of patients enrolled was small, however, this trend was still obvious. Through further cell experiments, we also found that low expression of GAS5 promoted proliferation and inhibited apoptosis of bladder cancer cells. Overexpression of miR‐21 promoted bladder cell proliferation and inhibited cell apoptosis. It was also confirmed by miR and a prediction and dual‐luciferase reporter experiments that miR‐21 was a target of GAS5 in bladder cancer cells, and that GAS5 might regulate miR‐21.

Recent study has shown that GAS5 could inhibit the metastasis of bladder cancer by regulating the expression of chemokine (C‐C motif) ligand 1.^24^ Study of Bian has also shown that GAS5 promotes apoptosis by inhibiting EZH2 transcription in bladder cancer.^12^ Study has shown that hepatocellular carcinoma patients who had higher GAS5 level or lower miR‐21 level had longer survival times. Upregulation of miR‐21 expression levels largely attenuated the inhibitory effect of GAS5 on hepatocellular carcinoma cells migration and invasion.^19^ Study found that in lung cancer patients, low GAS5 expression levels was associated with poor clinicopathological features, and GAS5 might regulate cisplatin resistance via miR‐21 and PTEN.^25^ A recent study suggested that upregulation of miR‐21 may contribute to the progression of bladder cancer.^26^ However, it was also shown that some miRNAs could regulate GAS5 expression. In esophageal squamous cell carcinoma, miR‐196a can promote cell growth by inhibiting GAS5.^27^ Zhang's study has shown that GAS5 is negatively correlated with miR‐21 expression in breast cancer cells, and that miR‐21 could not only interact with tumor suppressor gene PTEN, but also has the function of targeting lncRNA GAS5.^28^

Few studies were conducted on the regulation of bladder cancer by GAS5 and miR‐21. To investigate the mechanism by which GAS5 regulates proliferation and apoptosis through miR‐21 in bladder cancer cells, we conducted further studies. It was confirmed by site prediction and dual luciferase reporter experiments that PTEN was a downstream target gene of miR‐21. As a tumor suppressor gene, mechanism of PTEN has been extensively studied. In bladder cancer, low expression of PTEN can promote the proliferation of bladder cancer cells and inhibit its apoptosis by promoting the expression of antiapoptotic proteins and the expressions of cell cycle‐associated factors. TCGA data also showed higher survival rates in patients with high PTEN expression. Further studies have shown that inhibition of miR‐21 expression can reverse the proliferative and antiapoptotic effects induced by GAS5 silencing. Silencing of miR‐21 also upregulated the expression levels of apoptotic proteins and upregulated the cycle‐associated proteins, suggesting that GAS5‐regulated apoptosis and cell cycle through miR‐21. Further studies have also shown that miR‐21 has no significant effect on GAS5 levels, and that GAS5 silencing significantly upregulates miR‐21 levels and inhibits PTEN levels. The inhibition of miR‐21 reversed the effect of GAS5 on miR‐21 and PTEN, suggesting that GAS5 might regulate PTEN to participate in the proliferation and apoptosis of bladder cancer cells through miR‐21. Study of Tao has found that GAS5 could regulate cardiac fibroblast fibrosis by targeting miR‐21 to regulate PTEN/ MMP‐2 signaling pathway.^29^ In pancreatic cancer, GAS5 inhibits metastasis of pancreatic cancer cells by modulating the miR‐32‐5p/PTEN axis.^30^ Animal experiments in this study also showed that GAS5 and miR‐21 acted as antitumors by inducing expression of GAS5.

In summary, high levels of GAS5 and low levels of miR‐21 might be associated with higher survival rate in bladder cancer. Moreover, GAS5 could exert antiproliferative and proapoptotic effects on bladder cancer cells through miR‐21 and PTEN, which provided new approaches for the treatment of bladder cancer.

## CONFLICT OF INTEREST

None declared.
